# Genome-wide association study of mammary gland tumors in Maltese dogs

**DOI:** 10.3389/fvets.2023.1255981

**Published:** 2023-10-04

**Authors:** Keon Kim, Jung Eun Song, Jae Beom Joo, Hyeon A Park, Chang Hyeon Choi, Chang Yun Je, Ock Kyu Kim, Sin Wook Park, Yoon Jung Do, Tai-Young Hur, Sang-Ik Park, Chang-Min Lee

**Affiliations:** ^1^Department of Veterinary Internal Medicine, College of Veterinary Medicine and BK21 FOUR Program, Chonnam National University, Gwangju, Republic of Korea; ^2^Gwangju Animal Medical Center, Gwangju, Republic of Korea; ^3^Division of Animal Diseases and Health, National Institute of Animal Science, Rural Development Administration, Wanju-gun, Republic of Korea; ^4^Department of Veterinary Pathology, College of Veterinary Medicine and BK21 FOUR Program, Chonnam National University, Gwangju, Republic of Korea

**Keywords:** canine, genome-wide association study, Maltese, mammary gland tumor, single-nucleotide polymorphism

## Abstract

**Background:**

A genome-wide association study (GWAS) is a valuable tool for investigating genetic and phenotypic variation in many diseases.

**Objective:**

The objective of this study was to identify variations in the genomes of Maltese dogs that are associated with the mammary gland tumor (MGT) phenotype and to assess the association between each biological condition and MGT phenotype in Maltese dogs.

**Methods:**

DNA was extracted from 22 tumor samples and 11 whole blood samples from dogs with MGTs. Genome-wide single-nucleotide polymorphism (SNP) genotyping was performed, and the top 20 SNPs associated with various conditions and genetic variations were mapped to their corresponding gene locations.

**Results:**

The genotyping process successfully identified 173,662 loci, with an overall genotype completion rate of 99.92%. Through the quality control analysis, 46,912 of these SNPs were excluded. Allelic tests were conducted to generate Manhattan plots, which showed several significant SNPs associated with MGT phenotype in intergenic region. The most prominent SNP, located within a region associated with transcription and linked to the malignancy grade of MGT, was identified on chromosome 5 (*p* = 0.00001) though there may be lack of statistical significance. Other SNPs were also found in several genes associated with oncogenesis, including TNFSF18, WDR3, ASIC5, STAR, and IL1RAP.

**Conclusion:**

To our knowledge, this is the first GWAS to analyze the genetic predisposition to MGT in Maltese dogs. Despite the limited number of cases, these analyzed data could provide the basis for further research on the genetic predisposition to MGTs in Maltese dogs.

## Introduction

Mammary gland tumors (MGTs) are the most common neoplastic disease in female dogs, and approximately 50% of MGTs are malignant (1–3). Genetic predisposition as well as other factors, such as hormonal influence, diet, and obesity, are known to contribute to the risk of developing MGTs (4). The breeds with an increased MGT risk can be variable according to each study and geographic location, but several breeds such as Maltese, Poodles, Yorkshire Terriers have been reported to have increased incidence of MGTs (5–7). Clinical staging of canine MGTs may be performed using three factors according to the World Health Organization staging system: (1) size evaluation, (2) assessment of regional lymph node involvement, and (3) identification of distant metastasis (8).

A genome-wide association study (GWAS) is a useful tool for understanding genetic and phenotypic variation. However, its usefulness is dependent on the level of linkage disequilibrium (LD) as only a limited number of markers can be genotyped (9, 10). In one study, canine LD was found to be 20–50 times more extensive than that in humans, indicating that fewer SNPs are required for genotype analysis in dogs than in humans (11). Furthermore, dogs with a history of inbreeding are estimated to show low levels of genetic variation and a high degree of LD within breeds (10, 12). Due to the small genetic variation, a homogenous origin within a single breed may allow for easier identification of risk factors for canine MGTs.

There are limited studies of genetic mutations involved in the development of canine MGTs. In one study, one single-nucleotide polymorphism (SNP) in the BRCA1/2 genes, in which mutations are known to be associated with an 84% increased risk of breast cancer in human medicine, was confirmed to be significantly associated with canine MGTs (1, 9). In another study, the significance of 10 genes associated with human breast cancer was analyzed in dogs with MGT (1). However, most of previous studies have been based on gene mutations in human breast cancer.

So far, only a limited number of GWAS studies have performed to examine the association between specific SNPs and the susceptibility to MGT in dogs. Melin et al. (13) were the first to conduct GWAS analysis in English Springer Spaniels and identified significant candidate genes for canine MGT development (13). Furthermore, more recently, Mucha et al. (14) conducted a preliminary GWAS study in female dogs with MGT, leading to the discovery of different significant SNPs and candidate genes compared to previous research (15). Therefore, further genetic analysis including other breeds needs to be performed at the whole-genome level to elucidate the genetic factors for the development of canine MGTs.

The purpose of the present study is to identify genomic variations in Maltese dogs that affect the development of canine MGT and to investigate the association of genetic polymorphisms with MGT conditions. To our knowledge, this study is the first GWAS of canine MGTs in Maltese dogs.

## Materials and methods

### Animals

Thirty-three Maltese dogs (22 in the MGT group and 11 controls) were included in the study. All cases and controls were selected from dogs referred to the veterinary medical teaching hospital of Chonnam National University and local animal hospitals between July 2021 and June 2022. The medical records, physical examinations, diagnostic imaging, and cytologic and histopathologic findings of all Maltese dogs were evaluated retrospectively. The criteria for inclusion as a control included healthy Maltese dogs without other neoplastic lesions confirmed by physical examination and diagnostic imaging. Furthermore, all patients with reproductive or hormonal disorders were excluded. The experimental design of the study was approved by the Chonnam National University Institutional Animal Care and Use Committee (approval No. CNU IACUC-YB-2021-70).

### Distribution of MGTs

The macroscopic distribution of MGTs was classified according to whether the tumors had a bilateral distribution (involvement of both sides of the mammary chain) or a unilateral distribution (involvement of one mammary chain). The evaluation of the distribution was based on physical examination records and photographs from clinical charts.

### Histopathological classification of MGTs

All histological evaluations of MGTs was performed by only one pathological specialist. In this study, as a condition for analysis, mammary tumors were classified into benign tumors and malignant tumors.

### Malignancy grade

The MGT group was classified histopathologically into benign tumors and malignant tumors, and among the malignant tumors, the patients who were diagnosed with carcinoma were graded by the “Elston and Ellis grading method” used in human medicine (16). The grades for the association analysis were benign, Grade 1, Grade 2, and Grade 3.

### Obesity

In the MGT group, the body condition score (BCS) system was used to distinguish between the obese and normal groups (17, 18). The obese group consisted of the dogs with MGTs with scores of 8 to 9 out of 9 based on the BCS system. The normal group was composed of the dogs with MGTs with scores of 4 to 6 out of 9 on the BCS system.

### Sample collection and DNA preparation

After surgical removal of tumors in the MGT group, neoplastic tissues were fixed in 10% neutralized formalin solution. The pathological material was processed with “Pathcentre” and embedded into paraffin. The 3-μm slices were prepared and stained with hematoxylin and eosin (HE). For DNA extraction, residual neoplastic tissues were soaked in a 1.5-ml microcentrifuge tube containing RNAlater. Tissues soaked in RNAlater were stored at −80°C until DNA extraction. Fasting venous blood samples were collected for genomic DNA extraction in the control group. A 1-mL aliquot of the sample was added to an ethylenediaminetetraacetic acid (EDTA) anticoagulant tube, and the tubes were maintained on ice during transport and stored at −80°C.

Genomic DNA was extracted from neoplastic tissues and EDTA-treated whole blood using commercial kits (QIAamp DNA Mini Kit and QIAamp DNA Blood Mini Kit; Qiagen, Germany) according to the manufacturer’s instructions. DNA concentrations were measured using a spectrometer. The A260/A280 ratio of all extracted samples was within the normal range of 1.7–1.9. The extracted DNA was stored at −20°C until genotyping of SNPs.

### Genome-wide SNP genotyping and association study

Genotyping of SNPs was conducted in a commercial laboratory using the BeadChip system (CanineHD BeadChip; Illumina, USA). DNA from 22 dogs with MGTs and 11 control dogs was analyzed. These samples were processed for 3 days using a reading system (InfiniumR HD Assay Ultra system; Illumina). According to the manufacturer’s recommendations, the preparation and quantification of DNA samples were performed. After that, the samples were amplified and incubated for 20 h. The next day, the amplified DNA was fragmented by using restriction enzymes, and the fragmented DNA was precipitated and resuspended. Prepared DNA samples were pipetted into the BeadChip and incubated at 48°C for 20 h for hybridization. On day 3, the hybridized samples were extended and stained using the BeadChip. Visualization and scanning of the stained samples were achieved using a commercially available platform (Illumina iScan platform; Illumina).

### Quality control

Specialized software (Genome Studio software; Illumina) was used to analyze the output from iScan. The samples from both the MGT and control groups were combined to optimize genotype clustering. Polymorphic genotype data for each group were analyzed separately to assess the levels of heterozygosity and deviation from the Hardy–Weinberg equilibrium (<0.01).

A total of 173,662 SNPs were found to have polymorphisms within the combined sample set, with greater than 99% of the SNPs included in acceptable genotype clusters. In our samples, SNPs with call rates below 95%, those that were monomorphic, and those with minor allele frequencies of less than 5% in our samples were excluded. We also excluded SNPs on the X chromosome due to allelic imbalance. Principal component analysis (PCA) was performed to determine if the distribution of the dog samples between the MGT and control groups was tendentious.

### Association and statistical analysis

We performed six association analyses of 126,750 SNPs using specialized program (Variation Suite v8 software; Golden Helix, USA). Statistical analysis on these SNPs was conducted, and a nominal value of *p* less than 0.05 was considered significant. Fisher’s exact tests were used to compare the proportions of samples with different characteristics such as MGT phenotype, distribution, histopathology, obesity, and spayed or not. We also used multivariate linear regressions to examine the relationships between different groups, including malignancy grade.

### Interpretation of GWAS results

In GWAS analysis, *p*-values are obtained by testing the association between each SNP and a trait. To determine the significance of these associations, a significance level is typically used, which is often derived from the Bonferroni correction, known as the genome-wide significance level. As we are using a number of SNPs in GWAS analysis, using a significance level of 0.05 for each individual test would increase the probability of Type I errors. To address this issue, Bonferroni correction was conducted by dividing the overall significance level by the total number of tests, denoted as *κ*. The adjusted significance level (*α*′) after Bonferroni correction is calculated as follows: α′ = α/κ. Here, α represents the overall significance level for the entire analysis, and κ corresponds to the number of tests conducted, which, in the context of GWAS, is equivalent to the number of SNPs used in the analysis. In this study, we used a total significance level of 0.05, and with 126,750 SNPs analyzed, we calculated the significance level for each test, resulting in a genome-wide significance level of *α*′ = 3.94×10^−7^. This adjusted significance level is commonly used in a single test during GWAS analysis to control for the multiple comparisons issue.

### Identification of SNP genes and markers

We obtained data on SNP loci from the Ensembl genome browser (European Bioinformatics Institute, Cambridge, U.K.) to identify genes associated with independent SNPs in each analysis. To predict how the variants may affect gene transcripts, we used gene annotations from the Ensembl database. Our findings revealed the location of variants, including coding sequences or introns of alternatively spliced gene transcripts.

## Results

### Case and association condition group

The study involved a total of 33 female Maltese dogs; of these, 11 were spayed, and 22 were intact. The average age was 10.3 ± 2.72 years. Table 1 summarizes the fundamental characteristics of the Maltese dogs. For the association study, six different condition groups were formed based on clinical manifestations of MGT, as shown in Table 2. One Maltese dog was excluded from the conditional analysis as any information regarding the MGT-associated condition could not be obtained except for neutering. Histopathological evaluation showed that all malignant tumors were classified as carcinomas. The benign group (*n* = 6) consisted of simple adenoma (*n* = 3), complex adenoma (*n* = 1), intraductal papillary adenoma (*n* = 1), and lobular hyperplasia (*n* = 1). The group of carcinoma grade 1 (*n* = 6) was composed of 4 cases of tubular carcinoma, 1 case of tubulopapillary carcinoma, and 1 case of *In situ* carcinoma. Within the carcinoma grade 2 group (*n* = 6), there were 3 cases of complex carcinoma, 1 case of tubulopapillary carcinoma, 1 case of carcinoma combined with malignant myoepithelioma, and 1 case of intraductal papillary carcinoma. The carcinoma grade 3 group (*n* = 3) included 1 case of tubular carcinoma, 1 case of carcinoma along with malignant myoepithelioma, and 1 case of complex carcinoma. To prepare for variation analysis, DNA was extracted from MGT tissue for the case group, while for the control group, DNA was extracted from leukocytes in whole blood samples.

**Table 1:** Characteristics of the dogs in the present study.

Variations	Female	Spayed female	Total
Age, mean ± S.D.	9.9 ± 2.73	11.1 ± 2.39	10.3 ± 2.68
Total number (%)	22 (66.7%)	11 (33.3%)	33 (100.0%)
MGT (%)	16 (72.7%)	6 (27.3%)	22 (100.0%)
Control (%)	6 (54.5%)	5 (45.5%)	11 (100.0%)

MGT, mammary gland tumor.

**Table 2:** Number of dogs for association studies classified as each condition associated with MGT.

Conditions	Variables	Number of dogs
MGT phenotype	MGT	22
	Control	11
Distribution	Bilateral	10
	Unilateral	11
Histopathology	Benign tumor	6
	Malignant tumor	15
Malignancy grade	Benign	6
	Carcinoma grade 1	6
	Carcinoma grade 2	6
	Carcinoma grade 3	3
Obesity	Obesity	3
	Normal	18
Neutering	Spayed	6
	Unspayed	16

MGT, mammary gland tumor.

### Principal component analysis

**Figure 1:**
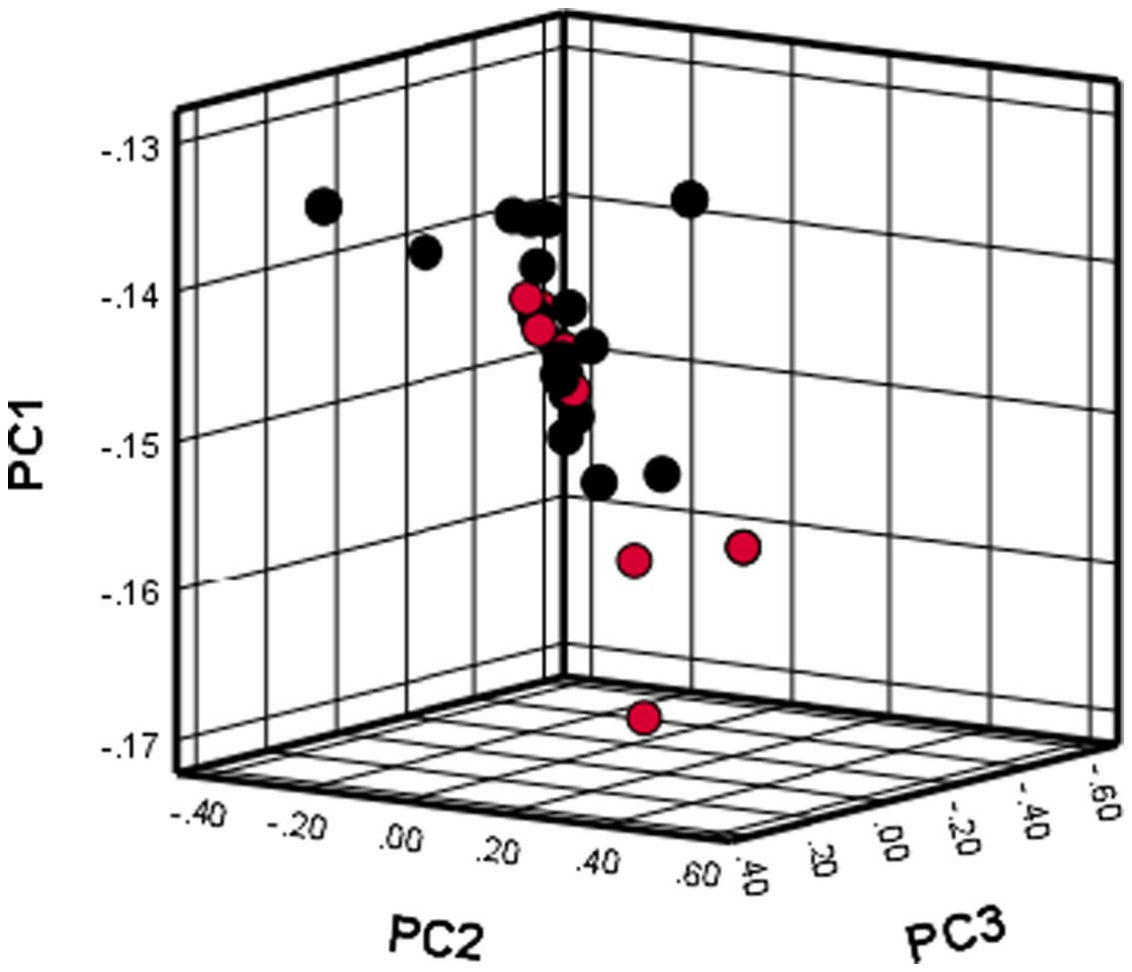
PCA results showing the distribution of cases and controls in the present study.

The use of PCA showed that all dogs included in the study were grouped together in one cluster, as illustrated in Figure 1. The results indicate that there was no population stratification within the sample.

### Genotyping result and association analysis

In this study, a total of 173,662 loci were genotyped in the samples, and of these, 46,912 SNPs were excluded, resulting in 126,750 SNPs that were used for subsequent analysis. The overall genotype completion rate was 99.92%.

**Figure 2:**
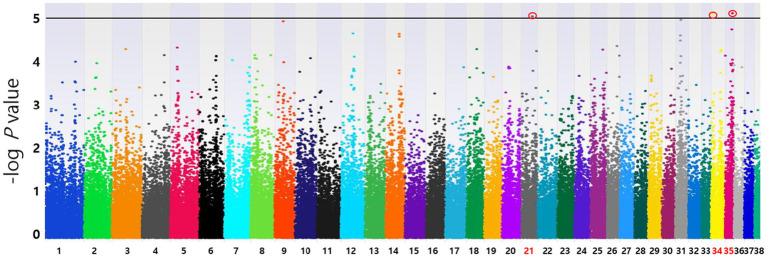
Manhattan plots of the GWAS for MGT phenotype. Fisher’s exact test was performed to confirm the statistical significance of SNPs. Chromosome numbers are displayed on the *X*-axis, and the results for each chromosome are shown in the diagram as individual colors for each chromosome. The *Y*-axis is the –log10 of the calculated *p*-values. Three significant SNPs associated with MGT phenotype were found in chromosome 21(*p* = 9.20×10^−6^), 34(*p* = 7.96×10^−6^), and 35(*p* = 7.81×10^−6^). All statistically significant SNPs have been marked with a red circle.

**Figure 3:**
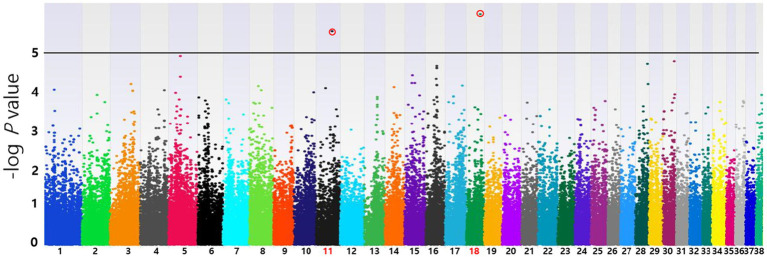
Manhattan plots of the GWAS for histopathological malignancy grade of MGT. Linear regression test was performed to confirm the statistical significance of SNPs. Chromosome numbers are displayed on the *X*-axis, and the results for each chromosome are shown in the diagram as individual colors for each chromosome. The *Y*-axis is the –log10 of the calculated *p*-values. Two significant SNPs associated with MGT phenotype were identified in chromosome 11(*p* = 2.39×10^−6^) and 18(*p* = 8.53×10^−7^). All statistically significant SNPs have been marked with a red circle.

Based on the SNP-chip results using the allelic test, Manhattan plots were produced to identify major loci associated with various conditions such as MGT phenotypes, macroscopic distribution of MGT, histopathological classification, malignancy grade, obesity, and neutering. The physical map position of the SNPs was plotted against the −log10 *p-*values. No SNPs that were significantly associated with MGT phenotype following Bonferroni correction were identified. However, a few significant peaks of association that reached the 5% threshold for genome-wide significance (10^−5^) were identified, as reported in a previous study (19). Three significant SNPs associated with MGT phenotype were found and all were located in the intergenic region (BICF2G630778783 in chromosome 35, BICF2P118387 in chromosome 34 and BICF2S23451480 in chromosome 21) (Figure 2). Similarly, two significant SNPs were identified for the malignancy grade of MGT (BICF2P660085 in chromosome 18 and BICF2S2324173 in chromosome 11) (Figure 3). Likewise, all these SNPs were located in the intergenic region. Although there may be no statistical significance, the most significant SNP which is located in transcription-related region as well as associated with MGT malignancy grade was found on chromosome 5 (*p* = 0.00001).

In this study, the top 20 SNPs based on their *p-*values were identified, and their corresponding gene regions were determined and are listed in Supplementary Tables S1–S6. The majority of SNPs for each condition were located in the intergenic region. Considering the difficulty in elucidating the roles of these SNPs during transcription and translation processes, we have excluded them from the table even if they meet the statistical significance criteria (*p* < 10^−5^). The SNPs with a high possibility of affecting gene transcription are summarized in Tables 3–5. Tables 3, 4 contain the GWAS results of Fisher’s exact tests for conditions that comprise two groups, while Table 5 presents the GWAS results of linear regression tests for conditions with more than two groups.

**Table 3:** Candidate SNP markers associated with the MGT phenotype.

Marker	Chromosome	Position	Gene	Variant	Allele (M > m)	MAF (Cases)	MAF (Control)	OR (95% CI)	*P-*value^†^	*P-*value^‡^	*P-*value^§^
BICF2S23741980	7	25,743,449	*TNFSF18*	Exon	A > T	0.024	0.273	0.00(0.00–0.30)	0.005	0.003	0.00009
BICF2S23750600	17	56,103,488	*WDR3*	Promoter	T > C	0.048	0.318	0.01(0.00–0.31)	0.006	0.003	0.0001
BICF2G63086613	7	78,537,371	*SEH1L*	Exon	C > A	0.262	0.773	0.11(0.02–0.50)	0.0002	0.002	0.0002
BICF2S23447362	25	40,378,887	*MFF*	Exon	C > T	0.143	0.682	0.12(0.03–0.54)	0.00004	0.001	0.0003
BICF2G630519109	14	4,790,093	*PLXNA4*	Exon	T > C	0.405	0.773	0.05(0.00–0.51)	0.008	0.004	0.0005

SNP, single-nucleotide polymorphism; MGT, mammary gland tumor; M > m, major allele to minor allele; MAF, minor allele frequency; OR, odds ratio; CI, confidence interval. ^†^Alleles; ^‡^Genotype; ^§^Linear regression. (Genome-wide significance level: *α*′ = 3.94×10^−7^).

**Table 4:** Candidate SNP markers associated with various conditions of MGT (distribution, histopathology, and neutering) derived from Fisher’s exact tests.

Condition	Marker	Chromosome	Position	Gene	Variant	Allele (M > m)	MAF (Cases)	MAF (Control)	OR (95% CI)	*P-*value^†^	*P-*value^‡^	*P-*value^§^
**Distribution**	TIGRP2P205207_rs8880787	15	54,080,059	*ASIC5*	Exon	A > G	0.2	0.545	0.00(0.00–0.41)	0.03	0.03	0.0001
	BICF2G630698889	18	35,416,737	*CCDC73*	Promoter	G > A	0.05	0.409	0.00(0.00–0.74)	0.01	0.01	0.0002
	BICF2G630378983	23	23,859,332	*EFHB*	Exon	T > C	0.15	0.636	0.01(0.00–1.20)	0.002	0.007	0.0003
**Histopathology**	BICF2P890779	37	15,124,213	*FAM237A*	Promoter	T > C	0.036	0.417	0.00(0.00–0.63)	0.006	0.002	0.0003
	BICF2G630848023	16	16,262,588	*SSPO*	Promoter	C > T	0.357	0.833	0.01(0.00–0.82)	0.01	0.02	0.0005
**Neutering**	BICF2G630111566	16	29,225,640	*STAR*	Exon	G > A	0.071	0.583	0.00(0.00–4.00)	0.001	0.007	0.0001

SNP, single-nucleotide polymorphism; MGT, mammary gland tumor; M > m, major allele to minor allele; MAF, minor allele frequency; OR, odds ratio; CI, confidence interval. ^†^Alleles; ^‡^Genotype; ^§^Linear regression. (Genome-wide significance level: α′ = 3.94×10^−7^).

**Table 5:** Candidate SNP markers associated with the malignancy grade of MGT derived from the linear regression test.

Marker	Chromosome	Position	Gene	Variant	Allele (M > m)	MAF	Major Homo (mean)	Hetero (mean)	Minor Homo (mean)	*P* value†	*R* value†	*P* value‡	Slope‡
BICF2S23436248	34	22,547,748	*IL1RAP*	Exon	A > G	0.385	9(2.00)	8(1.13)	4(0.00)	0.002	0.574	0.0002	−1.088
BICF2P605684	8	70,488,402	*MOK*	Exon	G > A	0.219	14(0.71)	5(2.40)	2(2.50)	0.003	0.558	0.0002	1.212

SNP, single-nucleotide polymorphism; MGT, mammary gland tumor; M > m, major allele to minor allele; MAF, minor allele frequency. ^†^Correlation/trend; ^‡^Linear regression. (Genome-wide significance level: *α*′ = 3.94×10^−7^).

## Discussion

Breeding practices of purebred dogs through selection pressure have led to decreased genetic variation within breeds (20). Due to this characteristic of dogs, they are being recognized as a model for studying various genetic diseases, and even a small number of gene analyses are valuable for evaluating their risks (21, 22). In this study, we selected Maltese dogs, which are known to be a breed at high risk of MGTs and are one of the most commonly bred dogs in South Korea (5–7). Canine MGTs and human breast cancer have many similarities in several aspects. Furthermore, since dogs spend considerable time with humans, they are very likely to be exposed to the same environmental factors that contribute to tumorigenesis (23). Considering these features, this study could establish a foundation for discovering candidate genes that may affect MGTs in dogs based on whole-genome analysis as well as extend research on breast cancer genes in humans.

Oncogenesis of MGTs is known to be multifactorial, but a high incidence of MGTs in specific dog breeds supports an important role of genetic factors (24). Several candidate genes, such as BRCA1, BRCA2, BRIP1, CHECK2, ERBB2, STK11, TOX3, TP53, and PTEN, have been reported to be associated with canine MGT risk based on previous research (1, 24). Furthermore, according to the first GWAS study on canine MGT by Melin et al. (13) the CDK5RAP2 gene exhibited a significant association in English Springer Spaniels (13). In this study, no SNP showed a significant association (*p* < 10^−5^) with the MGT phenotype in Maltese breeds except to located in intergenic region. As seen in the results, there is a possibility of the absence of statistically significant associations with SNPs, and there is also a likelihood that the results may have been affected by the small sample size. Therefore, further analysis with a larger sample size will be necessary in the future. Despite the results that no SNP showed association with the MGT phenotype, the most notable SNP, situated in a region related to transcription and linked to the malignancy grade of MGT, was discovered on intronic regions of candidate genes. The involvement of introns in regulation of tissue-specific gene expression, mRNA transcription, and translation has been well established (25). SNPs located in intron regions have the potential to generate splice variants of transcripts and promote or disrupt the function of long non-coding RNAs (lncRNAs) (26–28). Consequently, the genetic susceptibility to cancer could be influenced by SNPs located within introns (25).

In the MGT malignancy grade condition, genes such as MAP2K4, NRG1, and PPFIA2 were found to be associated with the identified SNPs (Supplementary Table S3). MAP2K4 is a member of the mitogen-activated protein kinase (MAPK) activator family in humans, and MAPK signaling is known to play a significant role in cell proliferation and differentiation (29). In cancer, MAP2K4 has the potential to activate phosphoinositide-3-kinase (PI3K)/AKT signaling, which can result in cell proliferation, migration, and invasion. Liu et al. (30) confirmed the possibility of MAP2K4 serving as an oncogene in breast cancer (30). NRG1 encodes ligands for the ERBB2-ERBB3 heterodimeric receptor tyrosine kinase, and gene fusions are believed to lead to autocrine receptor stimulation (31). ERBB2 is an important factor in breast cancer in human medicine. Patients with ERBB2-overexpressing breast cancer have lower overall survival rates and shorter disease-free intervals than patients whose cancer does not overexpress ERBB2. Moreover, ERBB2 overexpression has been linked to increased breast cancer metastasis (32). This study suggests that the genes related to tumor biology and the prognosis of breast cancer in humans may also be applicable to canine MGTs. The variations in the candidate genes that are associated with the MGT phenotype based on the tumor grade could ultimately affect their expression.

There were several SNPs that were found in important transcript sequences, which could have an impact on the structure and function of proteins. Of these SNPs, five were particularly significant in relation to the MGT phenotype and were found in transcripts of the TNFSF18, WDR3, SEHIL, MFF, and PLXNA4 genes (Table 3). TNF superfamily member 18 (TNFSF18) is known as common immune checkpoint gene. In the field of human medicine, blocking immune checkpoints has been shown to improve the immune response against cancer, and several agents targeting immune checkpoints have been identified and applied clinically to treat cancer. In addition, Li et al. (33) confirmed significant overexpression of TNFSF18 in breast carcinomas. Based on previous reports, the findings of this study suggest that TNFSF18, identified as a candidate gene, may have an expression abnormality associated with SNP variations in canine MGTs. This could also expand as another therapeutic option for MGTs in dogs, which are currently not actively targeted. WD repeat domain 3 (WDR3) has been shown to participate in different cellular processes including gene regulation, signal transduction, cell cycle progression, and apoptosis (34). Furthermore, the inhibition of WDR3 has been demonstrated to decrease breast carcinoma cell proliferation by McMahon et al. (35). Research on the correlation between WDR3 overexpression and the development of MGTs has not been conducted in both human and veterinary medical fields, but this study suggests a potential area for future research through its findings. SEH1-like nucleoporin (SEH1L) is associated with glycolysis, a complex reaction that initiates the catabolism of most carbohydrates (36). Cancer cells are characterized by the reprogramming of metabolism, and aerobic glycolysis is the primary energy source for these cells (37). In fact, it has been shown that the dysregulation of glycolysis occurs in several types of tumors (38–40).

The most significant SNPs associated with macroscopic distribution included ASIC5, CCDC73 and EFHB (Table 4). Acid-sensing ion channel (ASIC) is a member of the degenerin/epithelial Na + channel subfamily and is primarily expressed in the central and peripheral nervous system (41). The tumor microenvironment is typically acidic due to disorganized tumor vasculature, heterogeneous blood flow, and increased glycolysis in tumor cells (42). Previous studies have demonstrated that an acidic microenvironment contributes to breast tumor invasion and metastasis (43–45). In addition, Gupta et al. (41) suggest that ASIC1 contributes to breast cancer pathogenesis in response to acidic tumor microenvironments (41). Nonetheless, since the function of ASIC5 remains unknown, further studies are necessary to establish the specific characteristics in breast cancer. EFHB is a significant regulator of store-operated calcium entry (SOCE), a major mechanism that enables calcium entry from the extracellular region through the plasma membrane (45). SOCE supports a variety of breast cancer hallmarks, including cell viability, proliferation, migration, and metastasis (45).

One SNP that was located in the exon of the STAR gene was found to be among the most significant SNPs associated with neutering (Table 4). The STAR gene encodes the steroidogenic acute regulatory (STAR) protein, which plays a crucial role in the biosynthesis of steroid hormones by regulating the transportation of cholesterol, the substrate for all steroid hormones, from the outer to the inner mitochondrial membrane (46, 47). In humans, estrogens, particularly 17b-estradiol (E2), are known to promote breast cancer (48). Furthermore, dysfunction in the biosynthesis of androgens and/or estrogens has been linked to the development and growth of various cancers that are responsive to hormones (49). Manna et al. have confirmed that the STAR protein of MGT cells is expressed abundantly compared with normal mammary epithelial cells and that it is associated with increased levels of E2. It is possible that the abundant expression of STAR facilitates the delivery of cholesterol to the inner mitochondrial membrane, leading to the generation of additional precursors for E2, which could contribute to the promotion of breast tumorigenesis (48).

Obesity is known to be a risk factor for adverse effects of breast tumors due to numerous and complex reasons (50). However, in this study, significant SNPs were not identified according to the obesity conditions in the MGT group. Obesity could mainly be affected by acquired components such as environmental factors. However, this does not necessarily mean that genetic factors are not involved. Furthermore, only very few cases were classified as obese in this study.

In human breast cancer, it is known that the cancer can be classified into five molecular subtypes (Luminal A, Luminal B, Basal-like, ErbB2+, and Normal-like), and these classifications have been found to have significant associations with survival and clinical outcomes (51). When comparing this molecular subtype classification in human breast cancer to that of canine MGTs, it was confirmed that they share considerable similarities while there are a few differences (23). On that note, if the likelihood of developing a specific subtype of breast cancer is influenced by genetic factors, it would be anticipated that the novel discovered susceptibility genes exhibit varying levels of expression in each tumor subclasses. Additionally, it is expected that their transcription is regulated in cis by SNPs within them (52). In other words, some of the candidate genes associated with SNPs in crucial positions for transcription (identified through the GWAS study) may exhibit varying expressions across varying tumor subclasses. Therefore, it is important to remember that in future studies, the results of mRNA expression levels of these candidate genes should take into consideration the molecular subtypes.

The present study has several limitations. First, the study had a small sample size. This limited sample size may have had a restrictive effect on obtaining statistically significant results through Bonferroni correction. In addition, the possibility that the results might have been due to the small number of samples leading to the significant association between genomic variation and phenotype cannot be excluded. Second, as the GWAS analysis for confirming major SNPs associated with phenotype is based on the principle of common variant-common disease, next-generation sequencing (NGS) may be necessary for a rigorous whole-genome analysis. Finally, this study did not verify the identified candidate genes at the translation level. In this study, only functional predictions regarding candidate genes associated with MGTs were based on existing research. Further studies are needed to verify the expression of candidate genes that affect protein levels related to the tumorigenesis.

In conclusion, this study examined the genetic correlation between SNPs that are significantly linked to the MGT phenotype and various associated conditions. A number of genetic variations have been identified for the MGT phenotype and conditions, and the most significantly associated condition was the histological malignancy grade of MGT. To our knowledge, this is the first GWAS to analyze a genetic predisposition to the MGT phenotype and associated conditions in Maltese dogs. Despite the limited number of cases, all the analyzed data could be the basis for further research on the genetic predisposition to MGTs in Maltese dogs.

## Data availability statement

Original datasets are available in a publicly accessible repository: The original contributions presented in the study are publicly available. This data can be found here: https://www.ebi.ac.uk/eva/?eva-study=PRJEB65760.

## Ethics statement

The animal studies were approved by Chonnam National University Institutional Animal Care and Use Committee (approval No. CNU IACUC-YB-2021-70). The studies were conducted in accordance with the local legislation and institutional requirements. Written informed consent was obtained from the owners for the participation of their animals in this study.

## Author contributions

KK: Conceptualization, Data curation, Formal analysis, Investigation, Writing – original draft, Writing – review & editing. JS: Conceptualization, Resources, Writing – original draft, Writing – review & editing. JJ: Data curation, Resources, Writing – original draft. HP: Data curation, Visualization, Writing – original draft. CC: Data curation, Visualization, Writing – original draft. CJ: Data curation, Visualization, Writing – original draft. OK: Data curation, Project administration, Visualization, Writing – original draft. SP: Data curation, Project administration, Visualization, Writing – original draft. YD: Data curation, Funding acquisition, Visualization, Writing – review & editing. T-YH: Data curation, Funding acquisition, Visualization, Writing – review & editing. S-IP: Conceptualization, Data curation, Methodology, Resources, Supervision, Visualization, Writing – original draft, Writing – review & editing. C-ML: Conceptualization, Data curation, Funding acquisition, Methodology, Project administration, Supervision, Visualization, Writing – original draft, Writing – review & editing.
