# Supplementary material for: Genome-wide association study of mammary gland tumors in Maltese dogs
Source: Front Vet Sci. 2023 Oct 4;10:1255981. doi: 10.3389/fvets.2023.1255981 (PMC10583716; doi:10.3389/fvets.2023.1255981)
Supplement: Supplementary file 1 [file Table_2.docx]

Supplementary Material

Genome-wide association study of mammary gland tumors in Maltese dogs

Keon Kim^1†^, Jung Eun Song^1, 2†^, Jae Beom Joo^1^, Hyeon A Park^1^, Chang Hyeon Choi^1^, Chang Yun Je^1^, Ock Kyu Kim^1^, Sin Wook Park^1^, Yoon Jung Do^3^, Tai-Young Hur^3^, Sang-Ik Park^4*^ and Chang-Min Lee ^1*^

*** Correspondence: Sang-Ik Park and Chang-Min Lee**Corresponding Author
[sipark@jnu.ac.kr](mailto:sipark@jnu.ac.kr) and [cmlee1122@jnu.ac.kr](mailto:cmlee1122@jnu.ac.kr)

# Supplementary Tables

| Marker | Chromosome | Position | Gene | Variant* | Allele  (M>m) | MAF  (Cases) | MAF  (Control) | OR  (95% CI) | *P* value† | *P* value‡ | *P* value§ |
| --- | --- | --- | --- | --- | --- | --- | --- | --- | --- | --- | --- |
| BICF2S24412032 | 18 | 32589985 | *LDLRAD3* | Intron | A>G | 0.310 | 0.773 | 0.03(0.00-0.37) | 0.0006 | 0.0002 | 0.00005 |
| BICF2S23435953 | 8 | 62424591 | *CCDC88C* | Intron | C>T | 0.024 | 0.318 | 0.01(0.00-0.32) | 0.002 | 0.0007 | 0.00007 |
| BICF2P600734 | 35 | 23390471 | *CARMIL1* | Intron | C>A | 0.650 | 0.136 | 16.26(2.33-113.40) | 0.0001 | 0.0008 | 0.00009 |
| BICF2S23741980 | 7 | 25743449 | *TNLG2A* | Exon | A>T | 0.024 | 0.273 | 0.00(0.00-0.30) | 0.005 | 0.003 | 0.00009 |
| BICF2P1167028 | 9 | 28571473 | *CA10* | Intron | T>C | 0.071 | 0.591 | 0.06(0.01-0.43) | 0.00001 | 0.0006 | 0.0001 |
| BICF2P102405 | 5 | 22480585 | *FDX1* | Intron | A>G | 0.525 | 0.091 | 28.28(2.54-314.30) | 0.0008 | 0.005 | 0.0001 |
| BICF2S23750600 | 17 | 56103488 | *WDR3* | Promoter | T>C | 0.048 | 0.318 | 0.01(0.00-0.31) | 0.006 | 0.003 | 0.0001 |
| BICF2P1017405 | 18 | 32582786 | *LDLRAD3* | Intron | T>C | 0.333 | 0.773 | 0.04(0.00-0.41) | 0.001 | 0.002 | 0.0001 |
| BICF2G630232945 | 20 | 20519957 | *FOXP1* | Intron | C>T | 0.333 | 0.773 | 0.02(0.00-0.46) | 0.001 | 0.003 | 0.0001 |
| BICF2G630779898 | 35 | 23666416 | *CARMIL1* | Intron | C>T | 0.143 | 0.455 | 0.02(0.00-0.32) | 0.01 | 0.002 | 0.0002 |
| BICF2S23623001 | 14 | 44672737 | *PDE1C* | Intron | G>A | 0.262 | 0.727 | 0.09(0.02-0.45) | 0.0005 | 0.007 | 0.0002 |
| BICF2G630778876 | 35 | 22694176 | *ALDH5A* | Intron | A>G | 0.452 | 0.045 | 26.95(1.93-377.20) | 0.0006 | 0.009 | 0.0002 |
| TIGRP2P103421_rs8766755 | 7 | 73110339 | *ARHGAP28* | Intron | A>G | 0.452 | 0.045 | 23.84(1.96-290.31) | 0.0006 | 0.009 | 0.0002 |
| TIGRP2P193581_rs9095747 | 14 | 43691421 | *ADCYAP1R1* | Intron | C>T | 0.476 | 0.091 | 22.98(2.91-181.29) | 0.002 | 0.002 | 0.0002 |
| TIGRP2P163210_rs8729302 | 12 | 33135142 | *COL19A1* | Intron | C>T | 0.167 | 0.500 | 0.04(0.00-0.38) | 0.008 | 0.003 | 0.0002 |
| BICF2G630507328 | 24 | 14229081 | *PLCB1* | Intron | C>T | 0.310 | 0.773 | 0.07(0.01-0.49) | 0.0006 | 0.002 | 0.0002 |
| BICF2G63086547 | 7 | 78444458 | *SEH1L* | Intron | C>T | 0.262 | 0.773 | 0.11(0.02-0.50) | 0.0001 | 0.002 | 0.0002 |
| TIGRP2P105304_rs8695798 | 7 | 78453416 | *SEH1L* | Intron | G>A | 0.262 | 0.773 | 0.11(0.02-0.50) | 0.0001 | 0.002 | 0.0002 |
| BICF2G63086607 | 7 | 78531301 | *SEH1L* | Intron | T>C | 0.262 | 0.773 | 0.11(0.02-0.50) | 0.0001 | 0.002 | 0.0002 |
| BICF2G63086613 | 7 | 78537371 | *SEH1L* | Exon | C>A | 0.262 | 0.773 | 0.11(0.02-0.50) | 0.0001 | 0.002 | 0.0002 |

**Supplementary Table 1.** *P* values and association parameters for the top 20 SNPs and their gene regions in the GWAS of MGT phenotype in ascending order of allelic *p* values

**Supplementary Table 2.** *P* values and association parameters for the top 20 SNPs and their gene regions in the GWAS of macroscopic MGT distribution in ascending order of allelic *p* values

| Marker | Chromosome | Position | Gene | Variant* | Allele  (M>m) | MAF  (Cases) | MAF  (Control) | OR  (95% CI) | *P* value† | *P* value‡ | *P* value§ |
| --- | --- | --- | --- | --- | --- | --- | --- | --- | --- | --- | --- |
| BICF2P1266312 | 5 | 74295310 | C5H11orf53 | Intron | T>C | 0.050 | 0.455 | 0.00(0.00-0.90) | 0.004 | 0.0003 | 0.00004 |
| BICF2P433434 | 5 | 73419487 | WWOX | Intron | T>C | 0.050 | 0.455 | 0.01(0.00-0.34) | 0.004 | 0.0003 | 0.00008 |
| BICF2S23612144 | 4 | 29179408 | ZMIZ1 | Intron | C>T | 0.100 | 0.545 | 0.00(0.00-0.40) | 0.003 | 0.003 | 0.0001 |
| TIGRP2P205207_rs8880787 | 15 | 54080059 | ASIC5 | Exon | A>G | 0.200 | 0.545 | 0.00(0.00-0.41) | 0.03 | 0.03 | 0.0001 |
| BICF2P1012908 | 17 | 15174058 | WDR35 | Intron | A>G | 0.100 | 0.455 | 0.00(0.00-0.88) | 0.02 | 0.01 | 0.0001 |
| BICF2G630431282 | 15 | 42959806 | STAB2 | Intron | A>G | 0.100 | 0.545 | 0.01(0.00-0.40) | 0.003 | 0.02 | 0.0001 |
| BICF2P768152 | 5 | 70427228 | PLCG2 | Intron | C>T | 0.050 | 0.455 | 0.01(0.00-0.26) | 0.004 | 0.0003 | 0.0002 |
| BICF2G630698848 | 18 | 35444216 | CCDC73 | Intron | A>G | 0.050 | 0.455 | 0.00(0.00-0.79) | 0.004 | 0.01 | 0.0002 |
| BICF2G630698889 | 18 | 35416737 | CCDC73 | Promoter | G>A | 0.050 | 0.409 | 0.00(0.00-0.74) | 0.01 | 0.01 | 0.0002 |
| BICF2S23729345 | 18 | 35578566 | EIF3M | Intron | A>C | 0.050 | 0.364 | 0.00(0.00-0.71) | 0.02 | 0.008 | 0.0002 |
| BICF2G630588752 | 32 | 23965949 | SLC39A8 | Intron | T>C | 0.600 | 0.091 | 29.87(1.92-465.37) | 0.0008 | 0.003 | 0.0002 |
| BICF2G630422564 | 15 | 56414049 | RXFP1 | Intron | C>T | 0.200 | 0.682 | 0.02(0.00-0.52) | 0.002 | 0.01 | 0.0003 |
| BICF2P284945 | 5 | 79005119 | ZFHX3 | Intron | G>A | 0.667 | 0.045 | 32.06(2.02-508.09) | 0.00003 | 0.0009 | 0.0003 |
| BICF2G630422783 | 15 | 56105987 | GASK1B | Intron | T>C | 0.200 | 0.636 | 0.01(0.00-0.47) | 0.006 | 0.02 | 0.0003 |
| BICF2S22922118 | 31 | 21257843 | ATP5PF | Intron | A>G | 0.150 | 0.455 | 0.00(0.00-0.51) | 0.05 | 0.05 | 0.0003 |
| BICF2P606007 | 18 | 48916080 | ANO1 | Intron | G>A | 0.250 | 0.636 | 0.01(0.00-0.69) | 0.02 | 0.04 | 0.0003 |
| BICF2G630378983 | 23 | 23859332 | EFHB | Exon | T>C | 0.150 | 0.636 | 0.01(0.00-1.20) | 0.002 | 0.007 | 0.0003 |
| BICF2P60706 | 2 | 22727265 | FRMD4A | Intron | T>C | 0.150 | 0.591 | 0.01(0.00-0.80) | 0.005 | 0.03 | 0.0003 |
| BICF2P1292637 | 2 | 22731454 | FRMD4A | Intron | G>A | 0.150 | 0.591 | 0.01(0.00-0.80) | 0.005 | 0.03 | 0.0003 |
| BICF2S2348065 | 5 | 82205748 | GFOD2 | Intron | T>C | 0.100 | 0.682 | 0.04(0.00-0.49) | 0.0001 | 0.00001 | 0.0003 |

**Supplementary Table 3.** *P* values and association parameters for the top 20 SNPs and their gene regions in the GWAS of histopathological classification of MGT in ascending order of allelic *p* values

| Marker | Chromosome | Position | Gene | Variant* | Allele  (M>m) | MAF  (Cases) | MAF  (Control) | OR  (95% CI) | *P* value† | *P* value‡ | *P* value§ |
| --- | --- | --- | --- | --- | --- | --- | --- | --- | --- | --- | --- |
| BICF2P1138443 | 34 | 20489782 | LPP | Intron | A>G | 0.393 | 0.833 | 0.00(0.00-4.80) | 0.02 | 0.02 | 0.0001 |
| BICF2P315872 | 2 | 77738659 | CDC42 | Intron | T>C | 0.036 | 0.500 | 0.00(0.00-0.78) | 0.001 | 0.01 | 0.0002 |
| TIGRP2P396542_rs9126057 | 34 | 22028071 | P3H2 | Intron | A>G | 0.357 | 0.833 | 0.00(0.00-1.66) | 0.01 | 0.02 | 0.0002 |
| BICF2S23334793 | 11 | 13988500 | CEP120 | Intron | C>T | 0.071 | 0.583 | 0.01(0.00-0.51) | 0.001 | 0.002 | 0.0003 |
| BICF2P542617 | 7 | 17944437 | NIBAN1 | Intron | C>T | 0.321 | 0.917 | 0.03(0.00-0.51) | 0.001 | 0.005 | 0.0003 |
| TIGRP2P1891_rs8826081 | 1 | 37745599 | GRM1 | Intron | G>A | 0.214 | 0.750 | 0.02(0.00-0.54) | 0.003 | 0.003 | 0.0003 |
| BICF2P671458 | 7 | 24273449 | RABGAP1L | Intron | C>G | 0.286 | 0.917 | 0.03(0.00-0.52) | 0.0003 | 0.003 | 0.0003 |
| BICF2P1287283 | 5 | 50699542 | FGGY | Intron | C>A | 0.179 | 0.583 | 0.00(0.00-0.57) | 0.02 | 0.005 | 0.0003 |
| BICF2P890779 | 37 | 15124213 | FAM237A | Promoter | T>C | 0.036 | 0.417 | 0.00(0.00-0.63) | 0.006 | 0.002 | 0.0003 |
| BICF2P434501 | 37 | 15128054 | FAM237A | Intron | T>C | 0.036 | 0.417 | 0.00(0.00-0.63) | 0.006 | 0.002 | 0.0003 |
| BICF2G630110916 | 16 | 28543067 | TACC1 | Intron | G>A | 0.107 | 0.667 | 0.03(0.00-0.46) | 0.0007 | 0.004 | 0.0004 |
| BICF2S23536290 | 38 | 11120096 | USH2A | Intron | C>T | 0.036 | 0.500 | 0.01(0.00-0.48) | 0.001 | 0.002 | 0.0005 |
| BICF2G630848023 | 16 | 16262588 | SSPO | Promoter | C>T | 0.357 | 0.833 | 0.01(0.00-0.82) | 0.01 | 0.02 | 0.0005 |
| BICF2P1257162 | 5 | 29695145 | ANGPTL5 | Intron | G>T | 0.250 | 0.833 | 0.05(0.00-0.57) | 0.001 | 0.007 | 0.0005 |
| BICF2S23221080 | 7 | 20371193 | ABL2 | Intron | G>A | 0.036 | 0.417 | 0.02(0.00-0.30) | 0.006 | 0.002 | 0.0006 |
| TIGRP2P205840_rs9065763 | 15 | 56865841 | RAPGEF2 | Intron | T>C | 0.038 | 0.333 | 0.00(0.00-1.20) | 0.03 | 0.02 | 0.0007 |
| BICF2S23713385 | 25 | 43523446 | ARMC9 | 3'downstream | G>A | 0.036 | 0.417 | 0.02(0.00-0.31) | 0.006 | 0.002 | 0.0007 |
| BICF2S23042067 | 5 | 57524086 | PLCH2 | Intron | G>A | 0.107 | 0.667 | 0.03(0.00-0.76) | 0.0007 | 0.004 | 0.0007 |
| BICF2P1259747 | 28 | 32583314 | PLEKHA1 | Intron | C>T | 0.250 | 0.833 | 0.03(0.00-0.81) | 0.001 | 0.0006 | 0.0007 |
| BICF2P1260976 | 5 | 57455237 | MORN1 | Intron | T>G | 0.143 | 0.667 | 0.02(0.00-1.36) | 0.002 | 0.01 | 0.0008 |

**Supplementary Table 4.** *P* values and association parameters for the top 20 SNPs and their gene regions in the GWAS of malignancy grade of MGTs in ascending order of linear regression *p* values

| Marker | Chromosome | Position | Gene | Variant* | Allele  (M>m) | MAF | Major Homo  (mean) | Hetero  (mean) | Minor Homo  (mean) | *P* value† | *R* value† | *P* value‡ | Slope‡ |
| --- | --- | --- | --- | --- | --- | --- | --- | --- | --- | --- | --- | --- | --- |
| BICF2P1373995 | 5 | 36341461 | *MAP2K4* | Intron | A>G | 0.427 | 8(0.25) | 9(1.67) | 4(2.50) | 0.0001 | 0.690 | 0.00001 | 1.169 |
| BICF2S23112103 | 16 | 34288763 | *NRG1* | Intron | A>G | 0.385 | 10(2.00) | 6(1.00) | 5(0.20) | 0.0002 | 0.668 | 0.00002 | -1.156 |
| BICF2S23612710 | 15 | 23918592 | *PPFIA2* | Intron | C>T | 0.292 | 9(2.00) | 9(1.00) | 3(0.00) | 0.0004 | 0.647 | 0.00003 | -1.337 |
| BICF2S23148359 | 15 | 23152407 | *PTPRC* | Intron | A>G | 0.277 | 9(1.89) | 9(0.89) | 2(0.00) | 0.0006 | 0.651 | 0.00006 | -1.564 |
| TIGRP2P362677_rs8985672 | 28 | 39130713 | *EBF3* | Intron | A>C | 0.229 | 10(2.10) | 10(0.60) | 1(0.00) | 0.0007 | 0.625 | 0.00006 | -1.451 |
| BICF2G630115232 | 16 | 34631782 | *NRG1* | Intron | T>C | 0.417 | 9(1.89) | 7(1.29) | 5(0.20) | 0.0007 | 0.621 | 0.00006 | -1.225 |
| BICF2P1018348 | 16 | 34696456 | *NRG1* | Intron | T>C | 0.406 | 9(1.89) | 7(1.29) | 5(0.20) | 0.0007 | 0.621 | 0.00006 | -1.225 |
| BICF2G630115344 | 16 | 34745909 | *NRG1* | Intron | G>A | 0.417 | 9(1.89) | 7(1.29) | 5(0.20) | 0.0007 | 0.621 | 0.00006 | -1.225 |
| BICF2P1407715 | 17 | 53162408 | *BCAS2* | Intron | G>A | 0.177 | 14(0.71) | 7(2.43) | 0 | 0.0007 | 0.620 | 0.00007 | 1.736 |
| TIGRP2P190775_rs8718350 | 14 | 29226385 | *DGKB* | Intron | A>G | 0.438 | 5(2.20) | 11(1.45) | 5(0.00) | 0.0008 | 0.616 | 0.00007 | -1.210 |
| BICF2P1285557 | 1 | 36382088 | *UTRN* | Intron | G>T | 0.198 | 13(0.69) | 7(2.14) | 1(3.00) | 0.001 | 0.609 | 0.00009 | 1.473 |
| BICF2S23351400 | 4 | 75713554 | *ENSCAFT* | Intron | A>C | 0.354 | 11(2.09) | 7(0.43) | 3(0.33) | 0.001 | 0.607 | 0.00009 | -1.149 |
| BICF2P947946 | 3 | 70064270 | *WDR1* | Intron | G>C | 0.260 | 12(1.92) | 5(0.80) | 4(0.00) | 0.001 | 0.606 | 0.00009 | -1.038 |
| BICF2S23534797 | 10 | 62427505 | *USP34* | Intron | A>G | 0.333 | 9(0.56) | 9(1.44) | 3(3.00) | 0.001 | 0.602 | 0.0001 | 1.149 |
| BICF2P959468 | 5 | 21055573 | *SDHD* | Intron | G>C | 0.323 | 8(2.25) | 7(0.86) | 6(0.50) | 0.001 | 0.601 | 0.0001 | -1.038 |
| BICF2P19251 | 2 | 46594956 | *PDE4D* | Intron | C>T | 0.156 | 11(0.55) | 10(2.10) | 0 | 0.001 | 0.595 | 0.0001 | 1.633 |
| BICF2S23414483 | 17 | 25804410 | *MEMO1* | Intron | C>T | 0.208 | 14(0.71) | 5(2.40) | 2(2.50) | 0.001 | 0.591 | 0.0001 | 1.247 |
| BICF2P735658 | 13 | 39936355 | *ATP8A1* | Intron | A>G | 0.292 | 8(2.25) | 11(0.82) | 2(0.00) | 0.001 | 0.589 | 0.0001 | -1.275 |
| BICF2G630115276 | 16 | 34663232 | *NRG1* | Intron | G>T | 0.375 | 9(1.89) | 8(1.25) | 4(0.00) | 0.001 | 0.588 | 0.0001 | -1.199 |
| BICF2P695305 | 6 | 2858111 | *AUTS2* | Intron | A>G | 0.260 | 7(2.29) | 13(0.85) | 1(0.00) | 0.001 | 0.588 | 0.0001 | -1.613 |

**Supplementary Table 5.** *P* values and association parameters for the top 20 SNPs and their gene regions in the GWAS of obesity in ascending order of allelic *p* values

| Marker | Chromosome | Position | Gene | Variant* | Allele  (M>m) | MAF  (Cases) | MAF  (Control) | OR  (95% CI) | *P* value† | *P* value‡ | *P* value§ |
| --- | --- | --- | --- | --- | --- | --- | --- | --- | --- | --- | --- |
| BICF2G630158419 | 25 | 48774595 | RBM44 | Intron | G>A | 0.167 | 0.618 | 0.00(0.00-4.20) | 0.07 | 0.07 | 0.001 |
| BICF2G630810029 | 6 | 7455167 | DTX2 | Intron | T>C | 0.167 | 0.500 | 0.00(0.00-1.40) | 0.2 | 0.2 | 0.003 |
| BICF2P159065 | 5 | 79113302 | ZFHX3 | Intron | A>G | 0.167 | 0.531 | 0.00(0.00-3.22) | 0.2 | 0.2 | 0.004 |
| BICF2S23528772 | 5 | 79127368 | ZFHX3 | Promoter | T>C | 0.167 | 0.531 | 0.00(0.00-3.22) | 0.2 | 0.2 | 0.004 |
| BICF2S2363880 | 33 | 30743070 | APOD | Intron | C>T | 0.833 | 0.147 | 16.56(0.89-307.10) | 0.002 | 0.002 | 0.005 |
| BICF2P762723 | 10 | 36092023 | LIMS1 | Intron | G>A | 0.167 | 0.529 | 0.00(0.00-1.35) | 0.2 | 0.2 | 0.005 |
| BICF2P745302 | 10 | 3483078 | SLC16A7 | Intron | A>C | 0.833 | 0.235 | 22.74(0.91-570.00) | 0.01 | 0.01 | 0.005 |
| TIGRP2P10850_rs8783641 | 1 | 82222431 | PRUNE2 | Intron | G>A | 0.833 | 0.353 | 26.16(0.98-700.19) | 0.07 | 0.07 | 0.006 |
| BICF2S23127796 | 1 | 119149637 | CHST8 | Intron | G>A | 0.833 | 0.235 | 21.28(0.94-483.69) | 0.01 | 0.01 | 0.006 |
| BICF2P1405652 | 24 | 27440917 | KIAA1755 | Intron | T>C | 0.167 | 0.529 | 0.00(0.00-1.39) | 0.2 | 0.2 | 0.006 |
| BICF2P698457 | 31 | 9680557 | ROBO2 | Intron | T>C | 0.167 | 0.588 | 0.02(0.00-1.42) | 0.08 | 0.08 | 0.006 |
| BICF2P259588 | 27 | 24992005 | C2CD5 | Intron | A>G | 0.833 | 0.176 | 20.29(0.81-506.72) | 0.004 | 0.004 | 0.006 |
| BICF2P801872 | 6 | 54620728 | ALG14 | Intron | G>A | 0.667 | 0.176 | 22.06(0.94-520.33) | 0.03 | 0.03 | 0.006 |
| BICF2G630237965 | 5 | 73656753 | VAT1L | Intron | C>G | 0.167 | 0.441 | 0.00(0.00-6.13) | 0.4 | 0.4 | 0.006 |
| BICF2S23718644 | 23 | 38582215 | XRN1 | Intron | G>C | 0.833 | 0.294 | 23.74(0.78-722.69) | 0.02 | 0.02 | 0.007 |
| BICF2G630730672 | 31 | 7803082 | ROBO1 | Intron | G>A | 0.167 | 0.529 | 0.02(0.00-1.38) | 0.2 | 0.2 | 0.007 |
| BICF2P1343455 | 1 | 86915211 | TRPM3 | Intron | A>G | 0.833 | 0.294 | 24.84(0.72-852.39) | 0.02 | 0.02 | 0.007 |
| BICF2S23542923 | 23 | 29267392 | CPNE4 | Intron | T>G | 0.833 | 0.206 | 12.86(1.12-147.29) | 0.006 | 0.006 | 0.007 |
| BICF2P664157 | 5 | 75033911 | CNTNAP4 | Intron | C>A | 0.833 | 0.206 | 13.42(1.12-160.12) | 0.006 | 0.006 | 0.007 |
| BICF2S2307368 | 6 | 62494997 | COL24A1 | Intron | C>T | 0.833 | 0.176 | 13.97(1.02-191.50) | 0.004 | 0.004 | 0.007 |

**Supplementary Table 6.** *P* values and association parameters for the top 20 SNPs and their gene regions in the GWAS of neutralization in ascending order of allelic *p* values

| Marker | Chromosome | Position | Gene | Variant* | Allele  (M>m) | MAF  (Cases) | MAF  (Control) | OR  (95% CI) | *P* value† | *P* value‡ | *P* value§ |
| --- | --- | --- | --- | --- | --- | --- | --- | --- | --- | --- | --- |
| BICF2P266946 | 2 | 60797631 | CES1 | Intron | A>G | 0.393 | 0.833 | 0.00(0.00-1.51) | 0.02 | 0.04 | 0.00006 |
| BICF2G630111552 | 16 | 29208590 | LSM1 | Intron | G>A | 0.036 | 0.583 | 0.00(0.00-2.79) | 0.0003 | 0.002 | 0.00006 |
| BICF2G630111566 | 16 | 29225640 | STAR | Exon | G>A | 0.071 | 0.583 | 0.00(0.00-4.00) | 0.001 | 0.007 | 0.0001 |
| BICF2P1172998 | 10 | 47306661 | LRPPRC | Intron | G>A | 0.179 | 0.750 | 0.02(0.00-0.66) | 0.001 | 0.007 | 0.0002 |
| TIGRP2P140794_rs8553661 | 10 | 47310278 | LRPPRC | Intron | A>G | 0.179 | 0.750 | 0.02(0.00-0.66) | 0.001 | 0.007 | 0.0002 |
| BICF2S23636519 | 26 | 20552226 | TFIP11 | Intron | T>C | 0.286 | 0.750 | 0.00(0.00-0.74) | 0.01 | 0.03 | 0.0002 |
| BICF2G630557815 | 7 | 35237083 | C1orf100 | Intron | T>C | 0.143 | 0.583 | 0.00(0.00-2.18) | 0.008 | 0.03 | 0.0002 |
| TIGRP2P280104_rs9095506 | 21 | 11250220 | GRM5 | Intron | A>G | 0.321 | 0.833 | 0.02(0.00-0.48) | 0.005 | 0.02 | 0.0003 |
| BICF2P1290697 | 10 | 51421608 | LHCGR | Intron | A>G | 0.107 | 0.500 | 0.00(0.00-1.31) | 0.01 | 0.02 | 0.0003 |
| BICF2P1038162 | 17 | 29822587 | VIT | Intron | A>C | 0.036 | 0.500 | 0.00(0.00-0.69) | 0.001 | 0.002 | 0.0003 |
| TIGRP2P231689_rs8737325 | 17 | 40983075 | DNAH6 | Intron | T>C | 0.107 | 0.583 | 0.00(0.00-3.23) | 0.003 | 0.009 | 0.0003 |
| BICF2G630128854 | 37 | 23408578 | SMARCAL1 | Exon | T>G | 0.357 | 0.750 | 0.01(0.00-0.50) | 0.04 | 0.08 | 0.0004 |
| BICF2P1338749 | 10 | 46684174 | THADA | Intron | C>A | 0.071 | 0.417 | 0.00(0.00-0.53) | 0.02 | 0.007 | 0.0004 |
| BICF2G630340312 | 3 | 42290409 | IGF1R | Intron | C>T | 0.107 | 0.500 | 0.00(0.00-1.95) | 0.01 | 0.02 | 0.0004 |
| BICF2P299311 | 1 | 87889913 | MAMDC2 | Intron | T>C | 0.357 | 0.833 | 0.01(0.00-2.03) | 0.01 | 0.02 | 0.0004 |
| BICF2P265323 | 22 | 46692364 | DZIP1 | Intron | T>A | 0.143 | 0.583 | 0.01(0.00-1.32) | 0.008 | 0.03 | 0.0004 |
| BICF2S2301586 | 17 | 35904264 | NPHP1 | Intron | T>C | 0.036 | 0.583 | 0.02(0.00-0.50) | 0.0003 | 0.002 | 0.0004 |
| BICF2G630340305 | 3 | 42277773 | IGF1R | Intron | G>A | 0.143 | 0.500 | 0.00(0.00-1.86) | 0.04 | 0.06 | 0.0005 |
| BICF2G630812471 | 16 | 39631263 | SGCZ | Intron | A>G | 0.036 | 0.417 | 0.01(0.00-0.35) | 0.006 | 0.002 | 0.0005 |
| BICF2S237626 | 1 | 101753910 | PEG3 | Intron | T>C | 0.286 | 0.833 | 0.01(0.00-1.11) | 0.002 | 0.01 | 0.0006 |
